# A Streamlined High Performance Liquid Chromatography with Tandem Mass Spectrometry Based Workflow for Rapid Screening of Cellular Accumulation of Small Molecules

**DOI:** 10.1002/cmdc.202500753

**Published:** 2025-12-08

**Authors:** Alina Metzen, Katharina Rox

**Affiliations:** ^1^ Department of Chemical Biology Helmholtz Centre for Infection Research (HZI) Inhoffenstraße 7 38124 Braunschweig Germany; ^2^ Partner site Hannover‐Braunschweig German Center for Infection Research (DZIF) Inhoffenstraße 7 38124 Braunschweig Germany

**Keywords:** accumulation, antiviral activity, cellular accumulation, drug discovery, intracellular target

## Abstract

Assessing if compounds with intracellular targets reach their site of action is crucial for success in drug development. Cell type‐specific uptake goes beyond permeability studies, typically mimicking crossing the gut, the lung, or the blood–brain barrier. A medium‐ to high‐throughput cellular accumulation protocol in 96‐well format is presented using six compounds, evaluating optimal conditions varying several parameters, such as incubation time, compound concentration, and extraction protocol. An optimized assay protocol for cellular accumulation of distinct chemical classes is a compromise: No one‐extraction‐protocol‐fits‐all exists; equally, some compounds need longer incubation periods to reach maximal intracellular concentration. Reliable high performance liquid chromatography with tandem mass spectrometry based quantification of cellular accumulation for all six compounds to the nM range is achieved with a short 1 h incubation. Intracellular concentrations per cell count are determined in A549^ACE+TMPRSS2^ cells, taking nonspecific binding into account. Hence, this approach adds valuable information during the pre‐screening of compounds with intracellular targets. Finally, optimal assay conditions are emphasized as essential for predicting activity in vitro and in vivo, based on biochemical information and intracellular concentrations. In summary, the workflow for cellular accumulation determination can serve two scenarios: 1) Pre‐selection of compounds for screening purposes or 2) systematic optimization of conditions to advance compounds with intracellular targets.

## Graphical abstract

This study uses six small‐molecule inhibitors to evaluate optimal cellular accumulation assay conditions in a 96‐well format with high performance liquid chromatography with tandem mass spectrometry detection. The obtained medium to high‐throughput workflow is suitable for reliably identifying cellular accumulation, valuable for preclinical selection of compounds with intracellular targets.

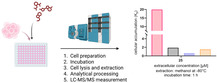

## 1 Introduction

During drug development, it is crucial to understand if a compound with an intracellular target is actually able to reach it. Unfortunately, strong target engagement, frequently assessed and reflected by biochemical assays, is not sufficient, as the cellular activity might be different, i.e., much lower. This “phenomenon” is often referred to as “cell drop‐off”.^[^ ^1^ ^]^ Several factors have been proven to contribute to this disconnect, such as insufficient cellular uptake or cellular accumulation,^[^ ^2^ ^]^ metabolism within the cell,^[^ ^3^ ^]^ binding to cellular proteins,^[^ ^4^ ^]^ or localization into cellular subcompartments.^[^ ^5^ ^]^ Therefore, in the recent decade, increasing effort has been undertaken to foresee the cellular bioactivity by linking the cellular bioavailability with biochemical activity to avoid failure because of missing activity in later stages of preclinical development.^[^ ^3^ ^,^ ^6^ ^,^ ^7^ ^]^

There are several ways for small molecules to be taken up. The most common one is passive diffusion, also highly dependent on a molecule's lipophilicity, as it enables a better interaction with the lipophilic cell membrane.^[^ ^8^ ^]^ Similarly, it has been shown that an increase in lipophilicity is positively correlated with a higher degree of passive diffusion into cells.^[^ ^5^ ^,^ ^8^ ^,^ ^9^ ^]^ However, different endocytic pathways might also be exploited by small molecules.^[^ ^9^, ^10^, ^11^, ^12^, ^13^, ^14^ ^]^ Equally, transporter‐mediated pathways, i.e., influx as well as efflux of compounds, can influence the intracellular bioavailability of a compound.^[^ ^10^, ^11^, ^12^, ^13^, ^14^, ^15^, ^16^, ^17^ ^]^

Several methods are conceivable for the determination of cellular accumulation.^[^ ^18^ ^]^ The majority of methods employ flow cytometric analysis (in particular for labeled macromolecules or nanoparticles),^[^ ^19^ ^]^ microscopic image analysis,^[^ ^20^ ^,^ ^21^ ^]^ radiolabeling of test compounds (especially for carrier‐mediated drug uptake),^[^ ^22^ ^,^ ^23^ ^]^ or label‐free mass spectrometric techniques.^[^ ^3^ ^,^ ^4^ ^,^ ^24^ ^]^ In general, the different published cellular accumulation assays to date rely on similar principles: Intracellular concentrations are determined by incubating a compound in 1) a specific cell line, with 2) a defined extracellular drug concentration for 3) a defined amount of time at 37 °C. After the respective incubation time, the supernatant and cellular fraction are separated to extract the intracellular fraction of the compounds after cell lysis.^[^ ^2^, ^3^, ^4^ ^,^ ^21^ ^,^ ^24^, ^25^, ^26^, ^27^, ^28^ ^]^ In particular, for cellular uptake studies involving drug transporters, also different ion conditions and inhibition by specific uptake inhibitors are investigated.^[^ ^29^, ^30^, ^31^ ^]^ Among the published studies, there is no consensus regarding cell lysis and extraction buffer. Typically, two to three wash cycles are followed by cell lysis and extraction of the compounds using acetonitrile:methanol (1:1),^[^ ^2^ ^]^ methanol,^[^ ^25^ ^,^ ^26^ ^]^ trypsin^[^ ^21^ ^,^ ^28^ ^]^ or a specific lysis buffer^[^ ^4^ ^,^ ^24^ ^,^ ^27^ ^]^ in combination with ultrasound,^[^ ^24^ ^,^ ^26^ ^]^ incubation at 95 °C^[^ ^25^ ^]^ or −30 °C^[^ ^27^ ^]^ and extraction at 4 °C overnight.^[^ ^2^ ^]^ In case of radiolabeling of test compounds, uptake and cellular accumulation is determined using liquid scintillation counting.^[^ ^23^ ^]^ Similarly, incubation time points for the determination of cellular accumulation vary as well. Studies assess intracellular concentrations after 2.5 h and up to 48 h; however, shorter incubation periods of 30–120 min have also been used.^[^ ^2^ ^,^ ^4^ ^,^ ^21^ ^,^ ^24^ ^,^ ^26^ ^,^ ^27^ ^]^ Uptake observed at 37 °C reflects the combined contribution of active transport, passive diffusion, and nonspecific binding, whereas at 4 °C, no active transport processes take place, and passive diffusion is minimal.^[^ ^3^ ^,^ ^32^ ^,^ ^33^ ^]^ Only a few studies take advantage of that fact and determine nonspecific binding of compounds to cellular membranes or assay plates at 4 °C instead of 37 °C.^[^ ^3^ ^,^ ^32^ ^,^ ^34^ ^]^ Finally, the majority of label‐free assays using mass spectrometry for readout are performed in larger formats, such as 24‐well^[^ ^4^ ^,^ ^26^ ^]^ and 6‐well plates^[^ ^21^ ^,^ ^24^ ^,^ ^27^ ^]^ or T‐25 cell culture flasks.^[^ ^3^ ^]^ A limited number of studies with high performance liquid chromatography with tandem mass spectrometry (HPLC‐MS/MS) detection are using 96‐well plates suitable for higher capacity, also to enable automation, whereas for radiolabeled compound detection 96‐well formats are more common.^[^ ^2^ ^,^ ^23^ ^,^ ^24^ ^,^ ^28^ ^,^ ^35^ ^]^ Larger assay formats require higher amounts of compound and are not suited for medium‐ to high‐throughput screening of cellular bioavailability. However, it is essential to recognize potential “cell drop‐offs”;^[^ ^1^ ^]^ in early drug development. In particular, in the drug development of anti‐infectives requiring BSL‐3 laboratories, not ubiquitously available, cellular accumulation determination can help to select the most promising candidates for evaluation under infection conditions.

In this study, we set out to establish a medium‐ to high‐throughput HPLC‐MS/MS‐based cellular accumulation assay in a 96‐well format using three standard compounds, i.e., verapamil, procainamide, and naproxen, as well as three antiviral compounds directed against SARS‐CoV‐2, i.e., remdesivir,^[^ ^36^ ^]^ 13b‐K,^[^ ^37^ ^]^ and nirmatrelvir.^[^ ^38^ ^]^ The aim of our study was to evaluate the impact of the different variables, i.e. extraction protocol, assay concentration, assay medium as well as incubation period, on the intracellular concentrations detected for different structural classes (peptidomimetics (13b‐K and nirmatrelvir), nucleosides (remdesivir), polar compounds acting on ion channels (procainamide),^[^ ^39^ ^]^ polar compounds with partially carrier‐mediated transport (naproxen)^[^ ^40^ ^]^ and P‐gp‐inhibiting nonpolar compounds (verapamil)). We selected for our study A549^hACE2+TMPRSS2^ cells^[^ ^41^ ^]^ as these are used for activity assessment of drugs against SARS‐CoV‐2. Exemplified by these different structural classes, we provide an optimized and robust workflow for rapid evaluation of novel drugs for intracellular concentration determination, which can be easily adapted to other cell lines, enabling to get a first estimation on the cellular accumulation potential of a compound.

## 2 Results

### 2.1 Investigation of Cellular Accumulation Conditions Reflecting Necessities of the Different Structural Classes

**Figure 1:**
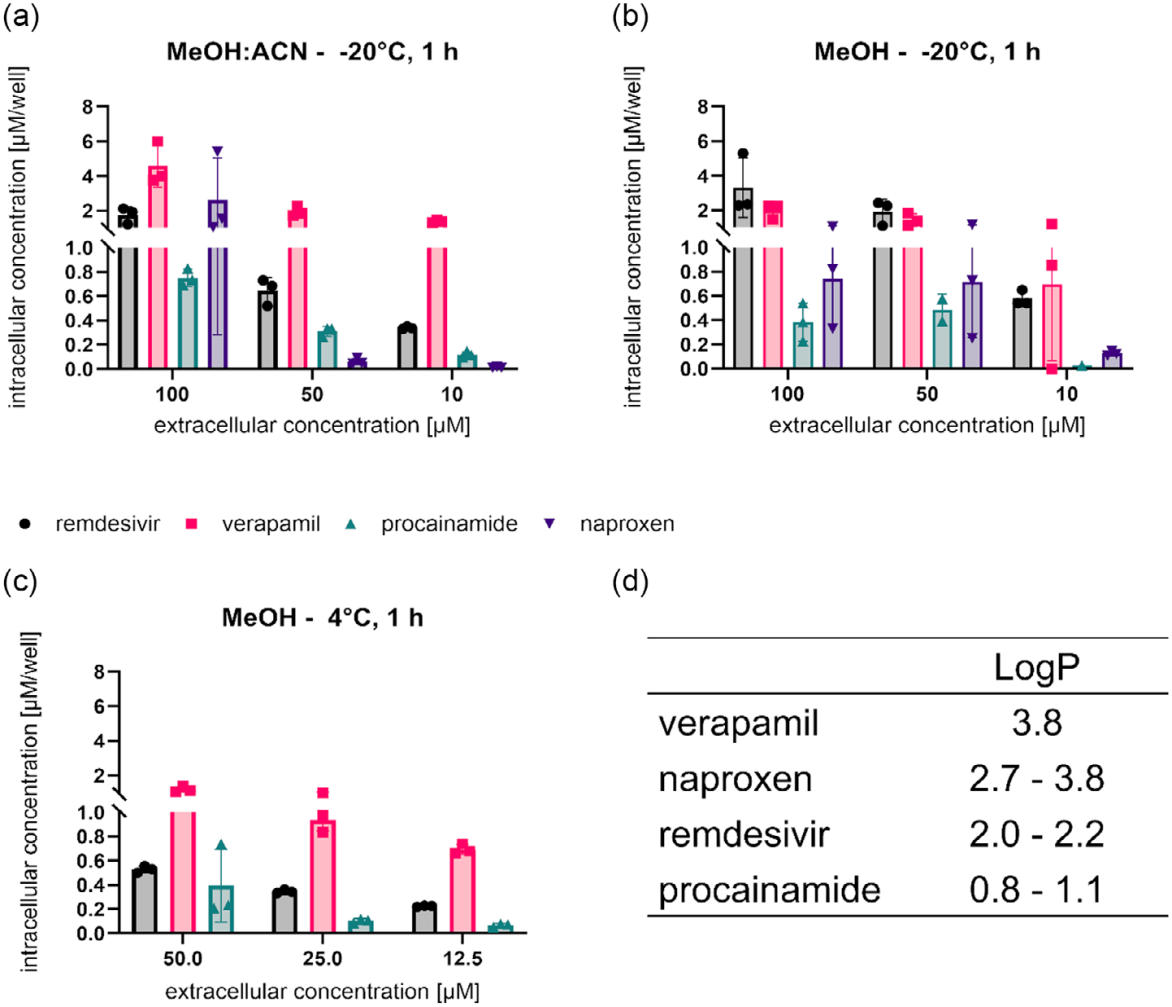
Cellular accumulation assays were performed with different extracellular concentrations and extraction conditions. Incubation of A549^hACE2+TMPRSS2^ cells using the four standard compounds remdesivir, verapamil, procainamide, and naproxen for 1 h. Compound concentrations of 10, 50, and 100 µM a,b) as well as 12.5, 25, or 50 µM c) were used. Substantial differences in intracellular concentration were found for all compounds between 100 µM extracellular concentration and 10 or 50 µM, as well as for 50 µM to 10 or 12.5 µM. However, the difference between 25 and 50 µM extracellular concentration was marginal. A 1:1 mixture of methanol:acetonitrile (a) was compared to methanol (b–c) as an extraction solvent for cell lysis at varying temperatures of 4 °C (c) to –20 °C (a,b). Three technical replicates were performed per assay. Literature logP values d) of the four test compounds are displayed for comparison of lipophilicity expressed as logP.^[^ ^44^, ^45^, ^46^, ^47^, ^48^, ^49^ ^]^

As a first starting point for the assay optimization, the compounds remdesivir, verapamil, procainamide, and naproxen were tested as a small reference test set using Dulbecco's Modified Eagle Medium (DMEM) + 2% fetal bovine serum (FBS) and an incubation time of 1 h. Cells were extracted with methanol at –20 °C overnight. Three different concentrations were used, i.e., 10, 50, and 100 µM. At the same time, the extraction conditions were varied. For the three concentrations, samples were either extracted using a mixture of methanol/ acetonitrile (1:1) (**Figure** **1**a) or methanol alone (Figure 1b). After extraction, both sample sets were incubated at –20 °C overnight. The intracellular concentration of remdesivir was not influenced by the extraction solvent, with around 2 µM of compound at an extracellular concentration of 100 µM for both conditions. For verapamil, the cellular accumulation values were doubled when using methanol/acetonitrile compared to methanol alone. For procainamide, this relationship was inverted so that methanol extraction resulted in increased intracellular concentrations, particularly observed at 50 µM extracellular concentration. Similar to verapamil, naproxen, as a lipophilic compound, resulted in higher intracellular concentrations using acetonitrile/methanol (Figure 1a,b). Remdesivir, verapamil, and procainamide showed the highest cellular accumulation values at 100 µM extracellular concentration, whereas naproxen had much lower values when using methanol as extraction solvent (Figure 1b). These relationships were not observed anymore when acetonitrile/methanol was used as extraction solvent (Figure 1a), highlighting the importance of careful selection of analytical post‐processing. For methanol as the extraction solvent, it was observed that intracellular concentrations were halved accordingly when only 50 µM as an extracellular concentration instead of 100 µM was used. At 10 µM extracellular concentration, procainamide was not well detected intracellularly; also, concentrations were increased for naproxen, verapamil, and remdesivir in a nonlinear manner compared to the 50 µM extracellular concentration (Figure 1b). Equally, for acetonitrile/methanol extraction, procainamide and naproxen were not detected at all or only at low concentrations when employing 50 or 10 µM as extracellular concentration. By contrast, a concentration‐dependent increase was observed for verapamil. For remdesivir, intracellular concentrations increased with increased extracellular concentrations; however, this relationship was nonlinear (Figure 1a). As methanol as extraction solvent served best to determine intracellular concentrations for all four tested compounds, albeit not being the best solution for extraction for every individual compound, we next varied the extraction temperature from –20 °C overnight to 4 °C for multiple hours. Moreover, because no benefit for the 100 µM concentration was seen over the 50 µM extracellular concentration, we fixed the upper concentration to 50 µM as a second variation of assay conditions. Furthermore, as intracellular procainamide was barely detectable after extraction with methanol at –20 °C overnight (Figure 1b), we fixed the lowest concentration to 12.5 µM for the assessment of the parameter extraction temperature. Additionally, we inserted an intermediate concentration of 25 µM. Using 4 °C for extraction resulted in the detection of reduced intracellular amounts for all four tested compounds. Strikingly, naproxen was not detectable anymore in any of the three different extracellular concentrations employed. Similar observations were made for procainamide, which was only detected intracellularly when using 50 µM as an extracellular concentration in substantial amounts compared to 10 and 12.5 µM. Also, for remdesivir, reduced intracellular concentrations were seen compared to the extraction with –20 °C. Only for verapamil, no impact of temperature for extraction was observed (Figure 1c). Thus, our results showed that the influence of the extraction temperature was substantially dependent on the chemical structure of the compounds. Hence, for further optimization of our protocol, we employed –20 °C as a fixed extraction temperature parameter.

**Figure 2:**
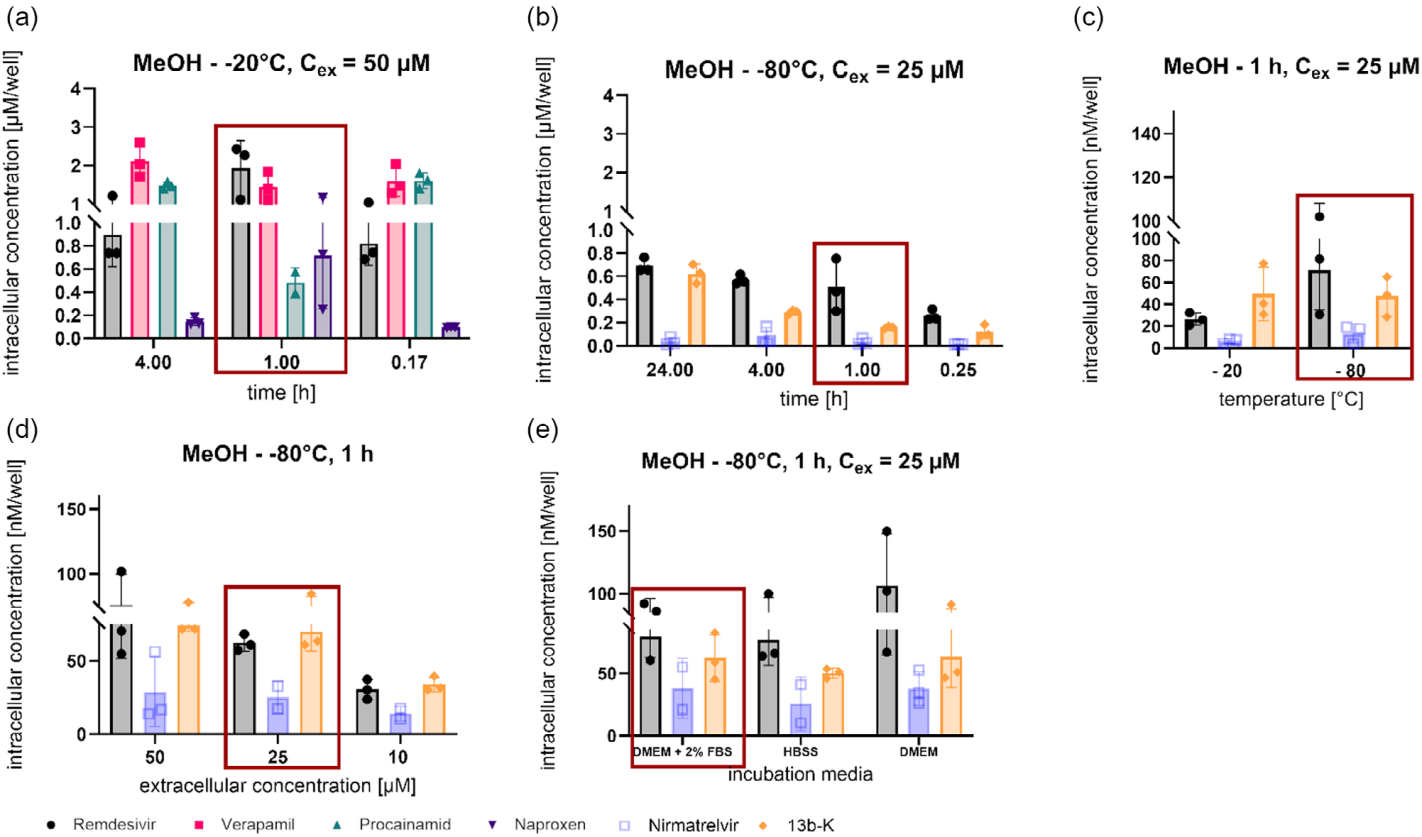
Intracellular concentrations at different incubation time points. The intracellular concentrations of remdesivir, verapamil, procainamide, and naproxen were tested at 10 min, 1, and 4 h a), whereas remdesivir and nirmatrelvir were evaluated at 15 min, 1, 4, and 24 h b). A549^hACE2+TMPRSS2^ cells were incubated with the respective compound at 50 µM (a) and 25 µM (b) for increasing incubation time points. The cell samples were thoroughly washed with PBS, lysed with methanol at –20 °C, and the sample concentration was determined by HPLC‐MS/MS. Optimal cell lysis temperature c), extracellular concentration d), as well as the incubation media e) were varied using remdesivir, nirmatrelvir, and 13b‐K. Red boxes represent the conditions used for the optimized workflow. Three technical replicates were performed per assay.

Initially, we had set the incubation period to 1 h to assess cellular accumulation. To examine the potential impact of incubation time, we selected first time points ranging from 10 min to 4 h for naproxen, verapamil, procainamide, and remdesivir. For all four compounds, similar intracellular concentrations were detected when incubating for either 10 min, 1 h, or 4 h. A slight decrease for procainamide was detected at 1 h, whereas a slight increase was seen for naproxen, potentially a result of variance in cell numbers (**Figure** **2**a). To examine incubation time points for other antiviral drugs targeting SARS‐CoV‐2, we also determined intracellular accumulation for 13b‐K and nirmatrelvir and kept remdesivir as comparator for the experiments with naproxen, verapamil, and procainamide. As we had not seen major differences for 50 and 25 µM as extracellular concentration, we set the extracellular concentration for this experiment to 25 µM. Because antiviral in vitro assays typically require at least incubation times of 24 h, we explored intracellular concentrations ranging from 15 min to 24 h. Intracellular concentrations slightly increased from 15 min to 24 h for remdesivir, which had been observed to a lesser extent for 50 µM extracellular concentration as well (Figure 2a,b). Equally, an increase in intracellular concentrations was detected for 13b‐K, whereas nirmatrelvir had substantially lower intracellular concentrations and equal concentrations were observed over time (Figure 2b). This finding underlines the importance for assessment of assessing the impact of incubation time, as some molecules might penetrate into cells more slowly. Whereas for in vitro assays it might be necessary to rather consider the incubation time point of 24 h for connecting antiviral with biochemical on‐target activity, the 1 h time point is more relevant for in vivo and a potential later clinical application. Because of compounds not persisting at high concentrations in, e.g., plasma over time, fluctuating concentration‐time profiles over time will be observed with less contact time for cellular accumulation. As a result, the 1 h incubation time point was set as the standard condition, bearing in mind that accumulation might be improved for longer contact times.

The influence of the extraction temperature had been set to –20 °C. However, also for the compounds 13b‐K and nirmatrelvir, we wanted to explore if intracellular concentrations can be improved further when lowering the extraction temperature even more. Therefore, we kept remdesivir as a reference to procainamide, naproxen, and verapamil from previous experiments, and added nirmatrelvir and 13b‐K at 25 µM for 1 h incubation. We extracted all compounds using methanol, but varied the temperature even further from –20 °C to −80 °C. Additionally, as we had noticed that washing with cold PBS might not remove the compound attached to the membrane, we changed the procedure to using pre‐warmed (37 °C) phosphate buffered saline (PBS) to remove residual membrane‐bound compound, which could cause bias in estimating intracellular concentrations. Our results showed that a higher intracellular concentration was detected when the extraction temperature was lowered to –80 °C overnight, even though the change was not substantial (Figure 2c). Moreover, we observed a reduced intracellular concentration for our comparator remdesivir, as expected, because the membrane‐bound compound was removed using pre‐warmed PBS. We took care of a short contact time during washing to avoid wash‐out of intracellular compounds. Next, we wanted to cross‐check the suitability of the 25 µM concentration for nirmatrelvir and 13b‐K as well. Therefore, remdesivir was used as a reference to link to the assays with procainamide, verapamil, and naproxen again. We chose three distinct concentrations ranging from 10 to 50 µM. It was observed that extracellular concentrations of 50 or 25 µM did not result in major differences in the respective intracellular concentration, suggesting that at 50 µM processes might have been already saturated for the chosen incubation period of 1 h. For the extracellular concentration of 10 µM, intracellular concentrations were around 2.5‐fold lower, thus linearity for cellular accumulation was observed (Figure 2d).

In general, several assay media are conceivable depending on the cell type used. In literature, medium supplemented with 2–10% FBS is frequently used for cellular accumulation assays,^[^ ^2^ ^,^ ^24^ ^,^ ^27^ ^,^ ^28^ ^]^ with some abolishing FBS completely by incubating only in Hank's balanced salt solution (HBSS)^[^ ^4^ ^,^ ^26^ ^]^ or PBS buffer.^[^ ^21^ ^]^ Therefore, we aimed to understand if assay media impacted intracellular concentrations. Thus, we used DMEM only, DMEM supplemented with 2% FBS, and HBSS buffer. Incubation with HBSS was performed once at 37 °C and 5% CO_2_, which are the conditions used for all assays performed with DMEM, and a second time at 37 °C while shaking, as some literature shake cells during cellular uptake assays.^[^ ^4^ ^,^ ^26^ ^]^ Independent of the assay medium, intracellular concentrations remained the same; only remdesivir had a higher standard deviation in DMEM, resulting in a higher mean intracellular uptake (Figure 2e). During that stage for assay optimization, we only assessed intracellular concentrations per well and did not yet normalize per cell number. This might explain the high variance observed, e.g., in the case of naproxen (Figure 1). Overall, the variance observed, even without normalization for 13b‐K, nirmatrelvir, remdesivir, verapamil, and for most of the conditions for procainamide, was low, enabling us to judge if conditions improved or worsened the assessment of uptake. For the next step, i.e., to judge cellular accumulation, we then employed normalization per cell number to enable accurate calculation of cellular accumulation.

### 2.2 Considering Unspecific Binding Effects to Avoid Bias

**Figure 3:**
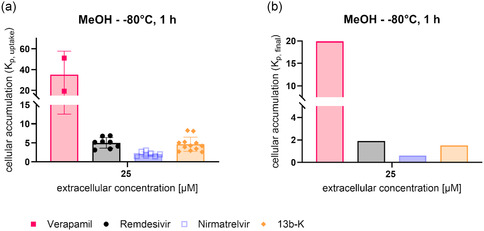
Cellular accumulation (K_p_) of verapamil, remdesivir, nirmatrelvir, and 13b‐K was determined using the final assay conditions a) as well as accounting for nonspecific binding to the assay plate b). For K_p,uptake_ (a), 2–12 biological replicates with 3 technical replicates per experiment are displayed per compound. K_p,nonspec,_ _binding_ was assessed in 2–3 biological replicates with 3 technical replicates each per compound. To receive K_p,final_, the average of all tested K_p,nonspec,binding_ values was subtracted from the average of all tested K_p,uptake_ values.

For the determination of cellular accumulation under the optimized assay conditions, the influence of nonspecific binding to the assay plate or cellular membranes was additionally taken into account . Therefore, the exact same protocol was used, but cells were incubated at 4 °C. For analysis, the detected compound concentration at 4 °C was subtracted from the intracellular concentration determined at 37 °C to obtain the final cellular accumulation values. As expected from the chemical structure, verapamil showed the highest intracellular concentration. Also, 13b‐K had a much higher intracellular concentration compared to nirmatrelvir (**Figure** **3**a). When taking inter alia the cell number per well into account, the cellular accumulation was calculated based on intracellular amounts per cellular volume and extracellular concentration as described previously.^[^ ^4^ ^]^ Remdesivir and 13b‐K showed intracellular accumulation with final K_p_ values of 1.9 and 2.2, respectively (Figure 3b). Nirmatrelvir, on the other hand, did not accumulate in A549^hACE2+TMPRSS2^ cells with a determined final K_p_ value of 0.6 (Figure 3b). Overall, the potential for intracellular accumulation was determined for four different compounds inheriting different structural properties.

**Scheme 1:**
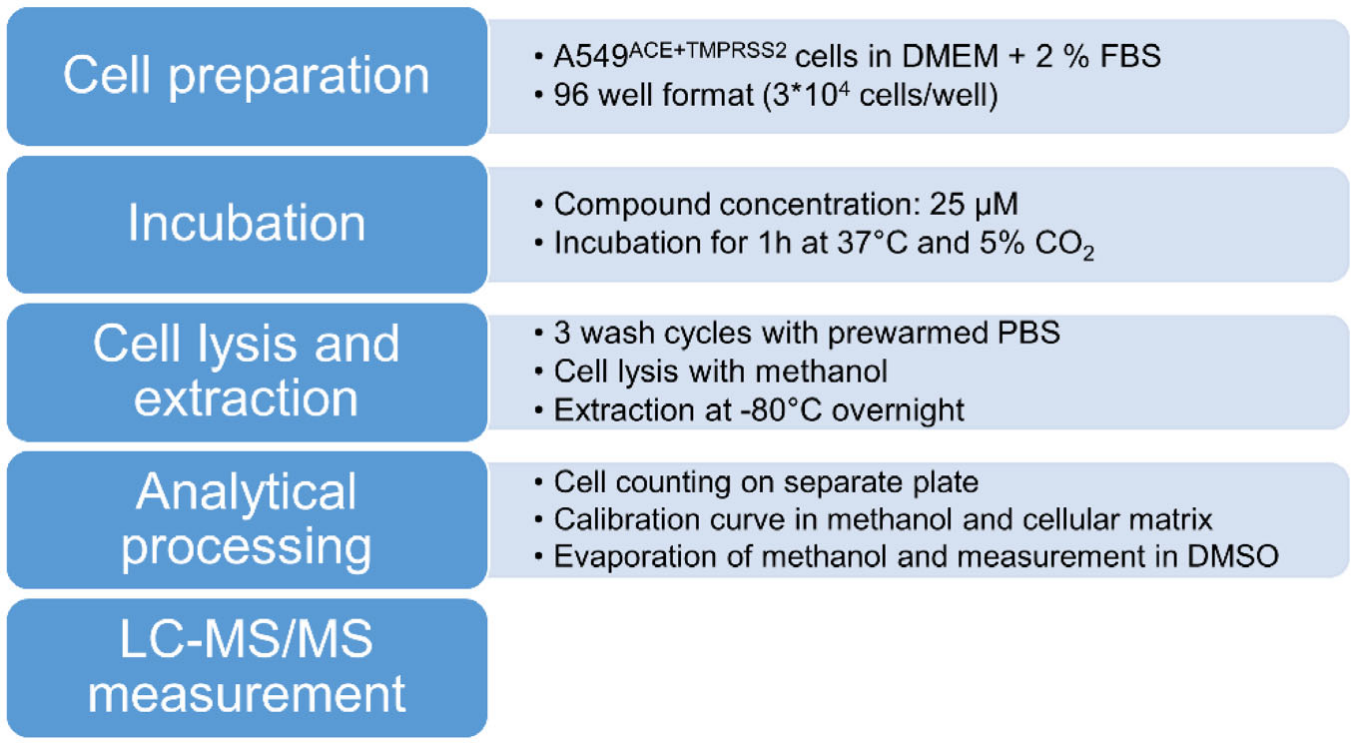
Optimized conditions with regard to cell preparation, compound concentration, incubation time, cell lysis and extraction, and analytical processing of the samples.

The final conditions for our streamlined HPLC‐MS/MS‐based assay for screening of cellular accumulation are depicted in **Scheme** **1**. We found that methanol served best as an extraction solvent for the different structural classes and that an extraction temperature of −80 °C overnight resulted in the detection of the highest intracellular concentrations. An extracellular assay concentration between 25–50 µM was needed to analytically detect intracellular concentrations for the limited number of structurally different compounds investigated herein. Furthermore, a 1 h incubation time allowed for rapid screening, avoiding to investigate complex interplays of cellular accumulation and compound stability issues during an initial drug development stage. Moreover, cell culture medium supplemented with only 2% FBS helped to keep cells viable and did not affect cellular accumulation. By varying the temperature of the assay from 37 °C to 4 °C, nonspecific binding was assessed so that unbiased intracellular uptake concentrations were obtained, and the intracellular accumulation was calculated. This is necessary information when targeting intracellular components and helps to advance during the drug development process.

## 3 Discussion

Cellular uptake and accumulation studies are important for early selection during preclinical development for compounds with intracellular targets. Especially for novel antivirals, cellular accumulation can bridge the gap between biochemical target activity and cellular antiviral assays, expediting the process without the need for specialized BSL‐2 or BSL‐3 facilities.^[^ ^7^ ^]^ Additionally, knowledge about the potential for cellular accumulation is also essential for the estimation of cytotoxicity and impact on cellular processes.^[^ ^1^ ^]^ Numerous studies have already been performed cellular accumulation experiments during preclinical development of novel drugs: Each study used different protocols for extraction, incubation times, and assay concentrations. Moreover, only a minority of studies deployed 96‐well formats^[^ ^2^ ^,^ ^24^ ^,^ ^28^ ^]^ and did not study conditions systematically with a particular focus on antivirals, expandable to other structural classes as presented for a limited number of compounds herein.

In this study, we developed a streamlined HPLC‐MS/MS‐based protocol serving to rapidly evaluate compounds for further development with a particular focus on antiviral compounds. We varied experimental conditions using six different compounds, among these three antivirals, i.e., remdesivir, nirmatrelvir, and 13b‐K. The drugs used to validate the assay were chosen based on molecular weight and polarity. Verapamil (454.6 g mol^−1^) and remdesivir (602.6 g mol^−1^), with higher molecular weight and more lipophilic structural elements, were expected to have higher intracellular concentrations than procainamide (235.3 g mol^−1^) and naproxen (230.3 g mol^−1^). Our study revealed that a protocol with equal conditions, as finally chosen for assessment of the six compounds in parallel, can help to get a first estimation of the cellular accumulation potential, but might not reflect the real intracellular accumulation, as this is highly dependent on the extraction protocol and assay conditions. Thus, it has to be differentiated if an assay is only employed for screening purposes, preferentially of the same chemical class, or if it shall weigh the intracellular concentrations of different chemical classes for selection. Our results show impressively that intracellular accumulation is underestimated, for, e.g., verapamil when methanol is used as the extraction reagent. We hypothesize that methanol is too hydrophilic to achieve a complete extraction of verapamil from the cells. On the contrary, the extraction solvent did not affect remdesivir presumably because of its amphiphilic properties. In general, our results for remdesivir are in line with previous observations showing accumulation of remdesivir in cells and terminal nerve membranes.^[^ ^26^ ^,^ ^42^ ^,^ ^43^ ^]^ The logP value serves as an indicator of lipophilicity. Thus, it would be expected to observe cellular accumulation in rank ascending order from procainamide^[^ ^44^ ^,^ ^45^ ^]^ < remdesivir^[^ ^46^ ^]^ < naproxen^[^ ^47^ ^,^ ^48^ ^]^ < verapamil^[^ ^49^ ^]^ (Figure 1d). However, our assessment revealed cellular accumulation in rank ascending order from procainamide ≤ naproxen < remdesivir < verapamil, suggesting additional mechanisms involved. This highlights the need to investigate intracellular accumulation, as intracellular concentrations cannot entirely be predicted from logP values, but might be a result of a combination of passive diffusion and carrier‐mediated transport. Also, for verapamil, a high cellular accumulation in fibrosarcoma cells has been observed. Moreover, verapamil was mainly found in lysosomes.^[^ ^50^ ^,^ ^51^ ^]^ Additionally, Berg and colleagues found that the intracellular concentration of certain photosensitizers was enhanced upon co‐treatment with verapamil.^[^ ^50^ ^]^ That aspect is interesting, but was not within the scope of the present study. Moreover, in this study, we were mainly interested in determining the overall intracellular concentration and did not distinguish into subcompartments, such as lysosomes. Takano et al. and Masago et al. studied the cellular accumulation of procainamide and revealed that it was saturable, temperature‐dependent, and increased when a basic pH in medium was used on the apical side of a polarized cell culture model. Overall, intracellular concentrations matched well with our assessments, even if they used fluorescence polarization for readout.^[^ ^39^ ^,^ ^52^ ^]^ In the study by Choi and colleagues, it was seen that naproxen had a concentration‐dependent cellular accumulation and inhibited monocarboxylic acid transporters.^[^ ^40^ ^]^ In a similar manner to the study with verapamil, we did not aim to study interactions, as this protocol shall be employed during early hit‐to‐lead drug discovery stages, although we are aware that the assessment of exact transporters involved in cellular uptake and accumulation is necessary at later stages of preclinical development.^[^ ^50^ ^]^ However, we think that both aspects raised by Berg et al. as well as Choi et al. are insightful, albeit not necessary for screening purposes for initial compound pre‐selection. Nirmatrelvir displayed the lowest accumulation of the three antiviral drugs, and equally low intracellular concentrations have been previously determined for the M^pro^ inhibitor by Higashi‐Kuwata and colleagues.^[^ ^25^ ^]^ So far, no cellular uptake or accumulation studies have been conducted for 13b‐K. However, initial pharmacokinetic studies with 13b‐K revealed high lung concentrations, which could be a result of high cellular accumulation.^[^ ^37^ ^]^

During the assessment of the optimal assay protocol, we just changed one assay condition after another while keeping other parameters constant. Apart from a significant influence of the extraction solvent on intracellular concentrations, extraction conditions, incubation periods, and the calibration matrix were important variables. For the incubation media, the FBS concentration is important, in particular for highly plasma protein‐bound drugs.^[^ ^2^ ^]^ Several published protocols rely on a reduced FBS concentration or even abolish FBS completely.^[^ ^2^ ^,^ ^4^ ^,^ ^21^ ^,^ ^26^ ^,^ ^28^ ^]^ Here, supplementation of cell culture medium with 2% FBS was chosen to ensure good cell viability and reduce the impact of high protein binding to assay medium. Moreover, as we selected a cell line used for antiviral assessments, we also aimed to render the assay comparable to antiviral activity studies in vitro. For this purpose, high extracellular compound concentrations of 10–100 µM were chosen to mimic conditions of in vitro antiviral activity studies. Higashi–Kuwata and colleagues selected similarly high concentrations for assessment of intracellular concentrations of nirmatrelvir and novel antivirals.^[^ ^25^ ^]^ Employing high compound concentrations might have limitations, especially when active carrier‐mediated drug transport is involved as, K_m_ values would be most likely exceeded. Additionally, the concentrations used in this study might exceed plasma concentrations in vivo, either. While these aspects are important to be considered during further drug development, they were not within the scope of this study, as we aimed to design the assay to bridge the gap from biochemical to cellular activity. We noticed a variety of different well formats for cellular accumulation assays in the literature. Typically, larger well formats of at least 24 wells are employed.^[^ ^4^ ^,^ ^21^ ^,^ ^24^ ^,^ ^26^ ^,^ ^27^ ^,^ ^53^ ^]^ Our assay was reliable in the 96‐well format, so that it enables screening of multiple compounds at the same time, allowing medium‐ to high‐throughput.

We found that an incubation period of 1 h worked best and was compatible for the purpose of medium‐ to high‐throughput screening. Equally, comparable cellular accumulation assays in the literature employ incubation periods of up to 2 h.^[^ ^4^ ^,^ ^21^ ^,^ ^26^ ^]^ However, we revealed that incubation periods for cellular accumulation assessments need to be compound‐specific if not used for screening and pre‐selection, but for calculating intracellular bioavailability as proposed by Mateus and colleagues, as well as Zhang and colleagues.^[^ ^4^ ^,^ ^26^ ^]^ If in vitro assay conditions for cellular accumulation do not properly reflect the maximum possible intracellular concentration, do not assess instabilities over time and differ in incubation periods typically used for in vitro activity assessment in cellular models, intracellular bioavailability might be under‐ or overestimated and, thus, bias calculations based on cellular unbound concentrations and biochemical on‐target activity. This might render correlations inaccurate.^[^ ^3^ ^,^ ^54^ ^]^ Similarly, in in vivo studies or later clinical application, compound concentrations in plasma might fluctuate, requiring an assay setup to reflect this if clinical efficacy shall be predicted based on in vitro data, as shown previously.^[^ ^26^ ^]^ As proposed for verapamil, distributing to lysosomes, it equally needs to be assessed if intracellular unbound concentrations reflect cytosolic concentrations or those in organelles only.^[^ ^50^ ^]^ To avoid membrane‐bound drugs as a confounding factor of cellular accumulation, the number of wash cycles was increased from two to three.

Complete cell lysis is essential for the detection of low intracellular concentrations as well as the reproducibility of the assay. Other studies employed temperature as a variable for extraction, e.g., –30 °C,^[^ ^27^ ^]^ 4 °C,^[^ ^2^ ^]^ and 95 °C^[^ ^25^ ^]^ were used in different protocols. We found that cell lysis can be further improved by incubation at –80 °C overnight. In contrast to the common method, i.e., to determine the total protein amount using Bradford or other methods to calculate the cell amount per well, we directly counted the cells of multiple assay wells at the start and the end of each experiment, making the protocol shorter and easier to apply. Furthermore, we determined that including a cellular matrix for the calibration curve improved the accuracy of the corresponding sample concentrations, as the cellular matrix for the calibration slightly quenched MS/MS signals. HPLC‐MS/MS measurement allows for accurate intracellular concentration determination down to a low nanomolar range, depending on the ionizability of the compounds. Additionally, no labeling was needed to detect the cellular accumulation. In the end, our data was handled in an equal manner for the calculation of intracellular accumulation as described previously,^[^ ^4^ ^,^ ^26^ ^]^ as both groups were similarly performing the cellular accumulation assays with the goal of correlating enzyme and antiviral activity of the tested compounds.

## 4 Conclusion

**Scheme 2:**
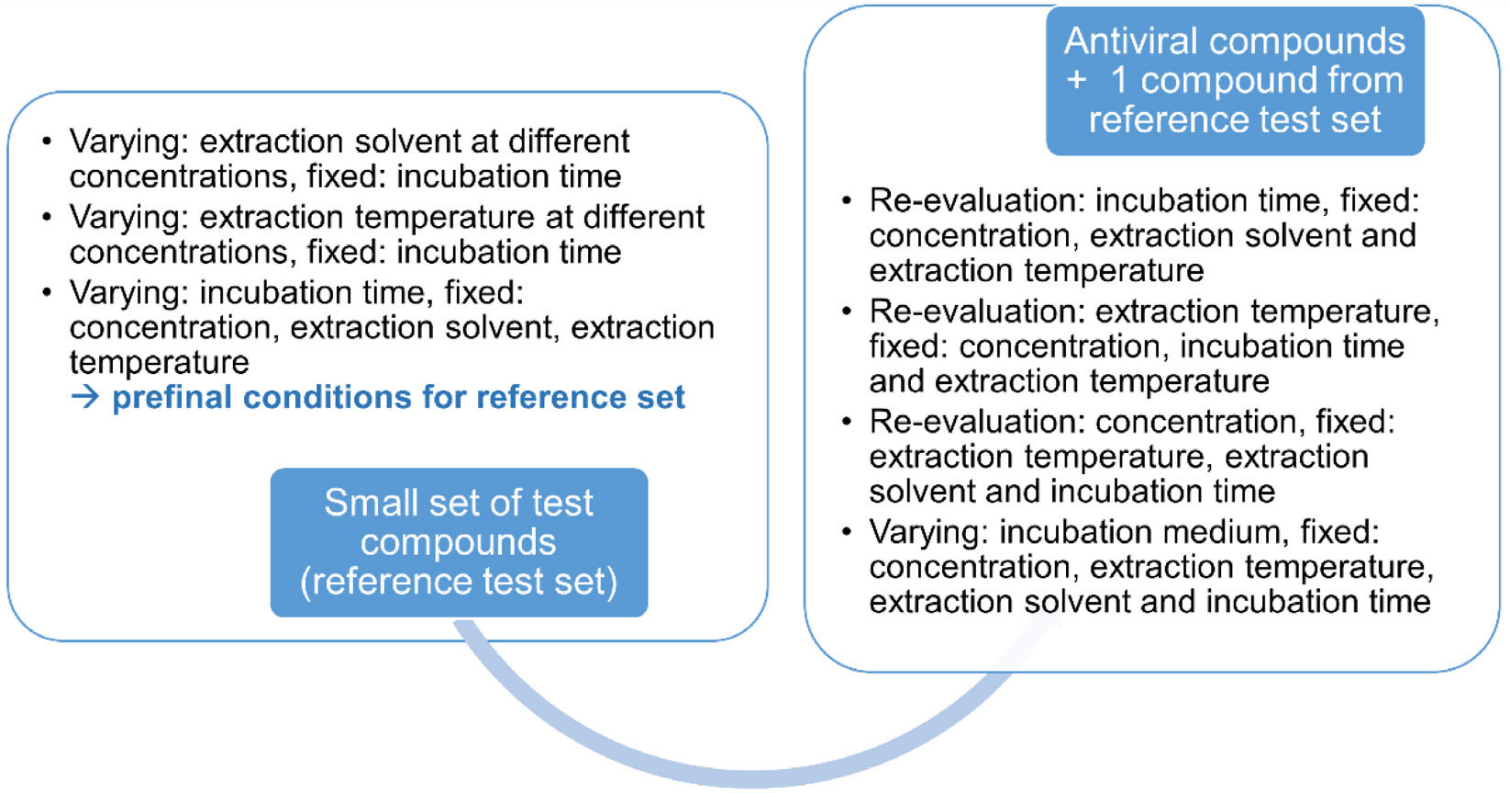
Systematics for optimization of parameters for the reference test set and the compound set, depicting which parameters were varied, fixed, and re‐evaluated.

In conclusion, we optimized cellular accumulation assay conditions with regard to incubation time, compound concentration, incubation media, extraction protocol, and analytical processing. First, we determined initial parameters for a small set of reference compounds, i.e., verapamil, naproxen, procainamide, and remdesivir, and then used this information to re‐evaluate the conditions for the antivirals, i.e., 13b‐K and nirmatrelvir, keeping remdesivir as a benchmark for previous assays with the small set of reference compounds (**Scheme** **2**). We found high‐throughput conditions with low extracellular concentrations and short incubation times, detecting the uptake of a variety of different drugs, also with low intracellular concentrations. Therefore, our cellular accumulation protocol is a useful tool to be included in preclinical compound selection for novel drugs. Finally, our study revealed that for accurate determination of intracellular bioavailability, a specifically adjusted protocol for distinct chemical classes is necessary to avoid confounding results when comparing structures derived from different chemical series. Here, our protocol revealed the most crucial parameters, i.e., extraction solvent and incubation time needed to provide a link for correlation of biochemical and in vitro cellular assays.

## 5 Experimental Section

5.1

5.1.1

##### Cell Lines and Compounds

A549^hACE2+TMPRSS2^ cells were obtained from the Goethe‐University Frankfurt^[^ ^41^ ^]^ and maintained in DMEM supplemented with 10% fetal bovine serum and 1% L‐glutamine (Gibco, 200 mM (100x), A29168−01).

13b‐K^[^ ^37^ ^,^ ^55^ ^]^ and Nirmatrelvir were provided by Prof Dr. Rolf Hilgenfeld from the University of Lübeck. Remdesivir was purchased from AdipoGen Life Sciences (CAS 1,809,249‐37−3). Verapamil hydrochloride (CAS 329,330,010) and procainamide hydrochloride (CAS 614‐39−1) were obtained from Acros Organics/ Thermo Scientific Chemicals, whereas (S)‐Naproxen (70290) was obtained from Cayman Chemical Company.

##### Cellular Accumulation Assay

A549^hACE2+TMPRSS2^ cells were seeded into 96‐well flat‐bottom tissue culture plates (Corning, #353075) at 3 × 10^5 ^cells ml^−1^ in DMEM + 2% FBS with a total volume of 100 µl/well. To ensure similar cell culture conditions and the same passage numbers, cells for the cellular accumulation assay and for cell counting, as well as the matrix source for the calibration curve, were seeded into three independent 96‐well cell culture plates at the same time. The cells were incubated overnight at 37 °C and 5% CO_2_. The following day, the cells were quickly washed with 37 °C pre‐warmed PBS once. Then, DMEM + 2% FBS containing the compounds at a final concentration of 25 µM was added at a final volume of 100 µl per well. In case of the cell counting plate and wells used as matrix for the calibration curve, 100 µl of DMEM + 2% FBS containing 0.5% DMSO instead of the compounds was added to the respective wells. All plates were incubated at 37 °C and 5% CO_2_ for 1 h. After incubation, the medium of each well containing compounds was transferred into a new 96‐well plate. The cells of all assay plates were quickly washed 3 times with 100 µl 37 °C pre‐warmed PBS per well in the final assay protocol. During the establishment of the protocol, assay plates were quickly washed 3 times with 100 µl cold PBS. For cell lysis, 100 µl of 4 °C cold methanol was added to each well and incubated overnight at –80 °C to ensure complete lysis. The following day, the methanol from the 96‐well cell plates was evaporated for analytical processing. After complete evaporation, 100 µl of methanol was added to each sample well of the assay plate. A calibration curve ranging from 0.1‐10,000 nM as well as quality control samples (QCs; concentrations 1, 10, 100, and 1000 nM) for the respective compounds (remdesivir, verapamil, procainamide, naproxen, nirmatrelvir, 13b‐K) were prepared in Eppendorf tubes by spiking the respective compound into 400 µl methanol, diluting it by twofold and mixed on an Eppendorf Mixmate vortex mixer for 5 min at 2000 rpm. 100 µl of methanol containing the calibration curve was added to each well with the cell matrix. The plates were mixed for 10 min at 800 rpm on an Eppendorf Mixmate vortex mixer. Next, 70 µl of each well was transferred into a 96‐well Greiner V‐bottom plate and centrifuged at 2276 × g and 4 °C for 20 min. The supernatants (50 µl per well) were transferred into new 96‐well Greiner V‐bottom plates. Then, methanol was evaporated again. After evaporation, 50 µl of DMSO was added to each well. The compound concentrations were determined using the calibration curve using HPLC‐MS/MS analysis as described in the section “HPLC‐MS analysis parameters”.

In addition to the final assay protocol, the following conditions were tested: A549^hACE2+TMPRSS2^ cells were seeded into 96‐well flat‐bottom tissue culture plates (Corning, #353075) at 2 × 10^5 ^cells ml^−1^ as well as 3 × 10^5 ^cells ml^−1^ in DMEM + 2% FBS with a total volume of 100 µl/well. In case 6‐well plates (Cellstar, Greiner bio‐one, cat.no. 657,160) were used, cells were seeded at a density of 3 × 10^5 ^cells ml^−1^ in DMEM + 2% FBS with a total volume of 2 ml/well. After solvent evaporation, 3 ml DMSO was added to each well, and 80 µl/well was transferred into a Greiner V 96‐well plate for centrifugation and HPLC‐MS/MS measurement. For 24‐well plates (Cellstar, #662160), A549^hACE2+TMPRSS2^ cells were seeded at a density of 2 × 10^5 ^cells ml^−1^ in DMEM + 2% FBS with a total volume of 0.6 ml/well, and 150 µl/well was used for analytical processing. Final compound concentrations of 10, 12.5, 25 and 50 µM were tested and incubated for the following time points: 10 min, 15 min, 1, 4 and 24 h, for cell lysis, 100 µl of either methanol or methanol:acetonitrile (1:1) were added to each well and incubated at 4 °C, –20 °C or –80 °C for 1–7 days. First, analytical processing of the cellular and supernatant fraction was performed simultaneously using only one calibration curve with a matrix consisting of DMEM + 2% FBS and the used extraction solvent in a 1:1 ratio. Later, only the cellular fraction was processed using methanol and cells as a matrix for all samples and the calibration curve.

##### Nonspecific Binding

Nonspecific binding to the assay plate or other components present during the experiment was tested in separate assays using the same compound concentrations, incubation time, and cell amount while incubating at 4 °C on an ice bath. All assay steps remained the same; however, the compound dilutions, incubation media, PBS, and the assay plate were cooled on ice for 15 min prior to the experiment. After incubation and removal of the incubation media, the cells were quickly washed 3 times with 100 µl of 37 °C pre‐warmed PBS per well to ensure equal wash conditions for the cellular accumulation experiments performed at 37 °C. Analytical processing remained identical to the previous section.

##### Cell Volume Determination

For each experiment, one 96‐well plate with A549^hACE2+TMPRSS2^ cells was used for the determination of the cell number on the assay day. Cells of two respective wells were counted once at the start of the assay and once after the respective incubation period of the experiment (vide supra). The cells were lysed with 100 µl/well trypsin at 37 °C and 2% CO_2_ for 2 min, transferred into an Eppendorf tube containing 200 µl of 37 °C warm DMEM + 2% FBS, and then washed twice with 100 µl DMEM + 2% FBS. The cell amount per well was counted using a Roche Innovatis Cedex XS cell counter. As A549‐cells have a round shape, V_cell_ was calculated as described previously^[^ ^26^ ^]^ with Equation (1):

(1) $$\begin{array}{c} V_{\text{cell}}=\frac{4\pi r^{3}}{3}*n \end{array}$$

where n is the number of cells counted as described. The diameter for the A549^hACE2+TMPRSS2^ cells was estimated to be 14 µm as described previously for A549 cells.^[^ ^26^ ^]^

##### Data Analysis

The mass spectrometric (MS), data were analyzed using Multiquant 3.0 software (AB Sciex, Darmstadt, Germany). Concentrations were determined by matching the peak area of the sample with the calibration curve. Each compound was tested in triplicate per assay. The graphs were created using GraphPad Prism 10. For the final assays, the ratio between the compound concentration per cellular volume and compound concentration in the medium, called cellular accumulation (K_p_), was calculated according to Equation (2):^[^ ^4^ ^,^ ^26^ ^]^

(2) $$\begin{array}{c} K_{p}=\frac{\frac{A_{\text{cell}}}{V_{\text{cell}}}}{C_{\text{medium}}} \end{array}$$

where A_cell_ is the measured intracellular concentration determined by cellular accumulation assays, C_medium_ is the extracellular concentration, and V_cell_ was calculated according to Equation (1). In case of assessment of nonspecific binding, the following equation was used to assess the final K_p_ value:

(3) $$\begin{array}{c} K_{\text{p,final}}=K_{\text{p,uptake}}\left[37{}^{\circ}C\right]-K_{\text{p,nonspec.}\;\text{binding}}\left[4{}^{\circ}C\right] \end{array}$$

where K_p,uptake_ is the average K_p_ value from all cellular accumulation assays performed at 37 °C, and K_p,nonspec. binding_ is the average K_p_ value from all assays performed at 4 °C.

##### HPLC‐MS/MS Analysis Conditions

An AB Sciex Qtrap6500plus mass spectrometer coupled to a 1290 Infinity II HPLC system (Agilent Technologies, Inc., Darmstadt, Germany) was used. The results were analyzed with the quantitation software MultiQuant 3.0 (AB Sciex, Darmstadt, Germany).

The liquid chromatography separation system with two binary pumps and a multisampler was used at a temperature of 30 °C, and the flow rate was set to 700 μl min^−1^. The column used was an Agilent Zorbax Eclipse Plus Reversed Phase C18 column with 50 × 2.1 mm length × diameter and a 1.8 μm particle size. The pre‐column used was an Agilent Zorbax SB C18 column with 30 × 2.1 mm length × diameter and a 3.5 μm particle size. Solvent A was 100% water + 0.1% formic acid, and solvent B was 95% acetonitrile + 5% water + 0.1% formic acid.

The following gradient was used for remdesivir, nirmatrelvir, and 13b‐K: 99% A until 0.1 min, 100‐0% A from 0.1 min until 4.0 min, 0% A until 4.5 min. The gradient for verapamil was as follows: 100% A until 1.0 min, 100‐0% A from 1.0 min until 5.0 min, 0% A until 6.0 min. For procainamide, the gradient was at 100% A until 0.5 min, 100‐0% A from 0.5 min until 3.0 min, 0% A until 3.5 min. Lastly, for naproxen it was: 99% A until 0.1 min, 99‐0% A from 0.1 min until 5.5 min, 0% A from 5.5 min until 6.0 min, 0‐99% A from 6 min until 6.4 min, and 99% A until 6.5 min. The injection volume was 10 μl per compound, and caffeine was used as an internal standard in all performed experiments.

The MS scan type was set as multiple reaction monitoring (MRM) with positive and negative mode, and the used MS‐parameters for the respective compounds are listed in Table S1, Supporting Information.

Additionally, an AB SCIEX Triple Quad^TM^ 7500 LC‐MS/MS system—QTRAP Ready mass spectrometer coupled to a 1290 Infinity II HPLC system (Agilent Technologies, Inc., Darmstadt, Germany) was used. The liquid chromatography separation system and the respective parameters are identical to the ones described previously.

The following gradient was used for remdesivir, nirmatrelvir, 13b‐K, verapamil, procainamide, and naproxen: 99% A until 0.5 min, 99‐0% A from 0.5 min until 1.0 min, 0% A from 1.0 min until 2.0 min, 0‐99% A from 2.0 min until 3.0 min, and 99% A until 4.7 min. The maximum pressure limit was set to 1300 bar. The injection volume was set to 5 µl.

The MS scan type was set as multiple reaction monitoring (MRM) with positive and negative mode, and the used MS‐parameters for the respective compounds are listed in Table S2, Supporting Information.

## Supporting Information

The Supporting Information contains supplemental tables S1 and S2 displaying MS/MS transitions.

## Conflict of Interest

The authors declare no conflict of interest.

## Author Contributions

**Alina Metzen** performed the experiments, analyzed the data, and wrote the original draft of the manuscript. **Katharina Rox** conceived, designed, and supervised the study, contributed to data analysis, and acquired funding. All authors reviewed and edited the manuscript and approved the final version of the manuscript.

## Supporting information

Supplementary Material

## Data Availability

The data that support the findings of this study are available from the corresponding author upon reasonable request.
