# Supplementary material for: A Streamlined High Performance Liquid Chromatography with Tandem Mass Spectrometry Based Workflow for Rapid Screening of Cellular Accumulation of Small Molecules
Source: ChemMedChem. 2025 Dec 8;21(4):e202500753. doi: 10.1002/cmdc.202500753 (PMC12913241; doi:10.1002/cmdc.202500753)
Supplement: Supplementary file 1 — Supplementary Material [file CMDC-21-e202500753-s001.pdf]

Supporting Information

A Streamlined HPLC-MS/MS-based Workflow for Rapid Screening of Cellular Accumulation of Small Molecules

Alina Metzen[a],[b] and Katharina Rox*[a],[b]

[a] A. Metzen, Dr. K. Rox
Department of Chemical Biology
Helmholtz Centre for Infection Research (HZI)
Inhoffenstraße 7, 38124 Braunschweig, Germany
E-mail: Katharina.rox@helmholtz-hzi.de

[b] A. Metzen, Dr. K. Rox
Partner site Hannover-Braunschweig
German Center for Infection Research (DZIF)
Inhoffenstraße 7, 38124 Braunschweig, Germany

Table of content

Table S1. Mass transitions of the internal standard, caffeine, and of the inhibitors remdesivir, nirmatrelvir, 13b-K, verapamil, procainamide and naproxen....................S2
Table S2. Mass transitions of the internal standard, caffeine, and of the inhibitors remdesivir, nirmatrelvir, 13b-K, verapamil, procainamide and naproxen....................S3

Table S1. Mass transitions of the internal standard, caffeine, and of the inhibitors remdesivir, nirmatrelvir, 13b-K, verapamil, procainamide and naproxen

ID	Q1 Mass (Da)	Q3 Mass (Da)	Time (msec)	DP (volts)	CE (volts)	CXP (volts)
Caffeine	195.024	138.000	20.0	80.000	25.000	14.000
Caffeine	195.024	110.000	20.0	80.000	31.000	18.000
Remdesivir	603.185	402.000	20.0	1.000	21.000	20.000
Remdesivir	603.185	228.900	20.0	1.000	25.000	26.000
Nirmatrelvir	500.146	319.100	20.0	131.000	21.000	22.000
Nirmatrelvir	500.146	110.100	20.0	131.000	27.000	14.000
Nirmatrelvir	497.974	471.000	20.0	-100.000	-26.000	-21.000
Nirmatrelvir	497.974	249.000	20.0	-100.000	-44.000	-21.000
13b-K	594.156	305.100	20.0	31.000	19.000	18.000
13b-K	594.156	249.000	20.0	31.000	35.000	20.000
13b-K	592.139	421.000	30.0	-155.000	-32.000	-21.000
13b-K	592.139	134.900	30.0	-155.000	-42.000	-15.000
Verapamil	454.688	165.000	30.0	1.000	35.000	28.000
Verapamil	454.688	303.100	30.0	1.000	35.000	18.000
Procainamide	235.744	163.000	30.0	80.000	21.000	18.000
Procainamide	235.744	120.000	30.0	80.000	39.000	12.000
Naproxen	231.106	185.100	30.0	80.000	19.000	10.000
Naproxen	231.106	170.200	30.0	80.000	33.000	12.000

Table S2. Mass transitions of the internal standard, caffeine, and of the inhibitors remdesivir, nirmatrelvir, 13b-K, verapamil, procainamide and naproxen

ID	Q1 Mass (Da)	Q3 Mass (Da)	Time (msec)	DP (volts)	CE (volts)	CXP (volts)
Caffeine	195.05	100.933	20	10	12	10
Caffeine	195.05	116.849	20	10	10	7
Remdesivir	603.17	199.982	20	10	52	11
Remdesivir	603.17	271.981	20	10	37	16
Remdesivir	603.17	402.134	20	10	24	20
Verapamil	455.31	165.038	30	10	39	15
Verapamil	455.31	303.197	30	10	37	26
Verapamil	455.31	150.015	30	10	59	8
Naproxen	231.23	185.049	30	10	23	29
Naproxen	231.23	170.049	30	10	38	21
Procainamide	236.2	163.049	30	10	37	10
Procainamide	236.2	119.998	30	10	31	12
Procainamide	236.2	92.011	30	10	58	11
Nirmatrelvir	498.204	471.285	30	-10	-28	-43
Nirmatrelvir	498.204	471.285	30	-10	-39	-23
13b-K	592.262	135.042	30	-10	-43	-14
13b-K	592.262	421.16	30	-10	-35	-32
13b-K	592.262	518.211	30	-10	-28	-14
